# Crystal structure of folliculin reveals a hidDENN function in genetically inherited renal cancer

**DOI:** 10.1098/rsob.120071

**Published:** 2012-08

**Authors:** Ravi K. Nookala, Lars Langemeyer, Angela Pacitto, Bernardo Ochoa-Montaño, Jane C. Donaldson, Beata K. Blaszczyk, Dimitri Y. Chirgadze, Francis A. Barr, J. Fernando Bazan, Tom L. Blundell

**Affiliations:** 1 Department of Biochemistry, University of Cambridge, Sanger Building, 80 Tennis Court Road, Cambridge CB2 1GA, UK; 2 Department of Biochemistry, University of Oxford, South Parks Road, Oxford OX1 3QU, UK; 3 NeuroScience, Inc., #373, 280th St., Osceola, WI 54020, USA

**Keywords:** Birt–Hogg–Dubé syndrome, folliculin, renal cell carcinoma, DENN

## Abstract

Mutations in the renal tumour suppressor protein, folliculin, lead to proliferative skin lesions, lung complications and renal cell carcinoma. Folliculin has been reported to interact with AMP-activated kinase, a key component of the mammalian target of rapamycin pathway. Most cancer-causing mutations lead to a carboxy-terminal truncation of folliculin, pointing to a functional importance of this domain in tumour suppression. We present here the crystal structure of folliculin carboxy-terminal domain and demonstrate that it is distantly related to differentially expressed in normal cells and neoplasia (DENN) domain proteins, a family of Rab guanine nucleotide exchange factors (GEFs). Using biochemical analysis, we show that folliculin has GEF activity, indicating that folliculin is probably a distantly related member of this class of Rab GEFs.

## 2. Introduction

Birt–Hogg–Dubé syndrome (BHD) is an inherited genetic disorder that predisposes individuals to renal cell carcinoma (RCC), benign skin tumours and lung cysts that lead to recurrent spontaneous pneumothorax [1,2]. Although BHD syndrome was first described in 1977 [3], it was not until 2002 that the gene encoding folliculin was identified and its mutation associated with the disease [1]. However, the cellular function of the protein remains unknown.

Folliculin and its interacting partners, FNIP1 and FNIP2, were shown to form a complex with AMP-activated protein kinase (AMPK) [5,6]. The involvement of folliculin, via AMPK, in mammalian target of rapamycin complex 1 (mTORC1) signalling remains unclear, as conflicting evidence has been reported [4,7–10]. Folliculin was also reported, in two separate studies, to be involved in the transcriptional regulation of proteins in the transforming growth factor β (TGF-β) pathway. In the first study, Cash *et al.* [8] showed apoptotic defects in *FLCN*-deficient cell lines as a direct result of downregulation of a transcription factor, *Bim*, which is involved in the TGF-β pathway. In the second study, Hong *et al.* [10] showed that several genes from the TGF-β pathway are differentially expressed in cells with and without folliculin. Additionally, Preston *et al.* [11] recently demonstrated that loss of folliculin increases transcriptional activity of hypoxia-inducing factor 1-α (Hif1-α), a phenomenon often seen in RCC. RCC is a complex type of genitourinary cancer with different tumour histologies [2,12], instances of which have increased in the past few decades, accounting for 2–3% of all adult cancers and more than 80% of kidney cancers [13,14]. So far, mutations in seven genes (folliculin, von Hippel-Lindau protein, the proto-oncogene MET, the TSC1 and TSC2 proteins of the tuberous sclerosis complex, fumarate hydratase and succinate dehydrogenase) have been associated with metabolic-disorder-related RCCs [2]. There is a correlation between the histological subtype of RCC and the causal gene mutation; however, interestingly, all histological subtypes have been reported in BHD patients [15].

In BHD patients, the most common germline mutation that can lead to RCCs occurs in the mutation hotspot, exon 11, of the *FLCN* gene, and produces a truncated folliculin protein that lacks the C-terminal half [16]. It is not known whether tumours from patients carrying these truncating mutations, or from any other identified mutations, express endogenous mutated folliculin. Intriguingly, while the C-terminal domain of folliculin is highly conserved in vertebrates, it is seemingly absent in the putative yeast orthologue, Lethal with Sec13 protein 7 (LST7; [17] and electronic supplementary material, figure S1). The LST7 protein was shown to have an involvement in regulating amino acid transport, through trafficking of the GAP1 general amino acid permease between the Golgi and plasma membrane [17].

In this study, we use structural and biochemical analyses to show that folliculin is probably a distant relative of the differentially expressed in normal cells and neoplasia (DENN) family of guanine nucleotide exchange factors (GEFs) and demonstrate that it possesses *in vitro* nucleotide exchange activity towards Rab35 GTPase.

## 3. Results and discussion

**Figure 1.**
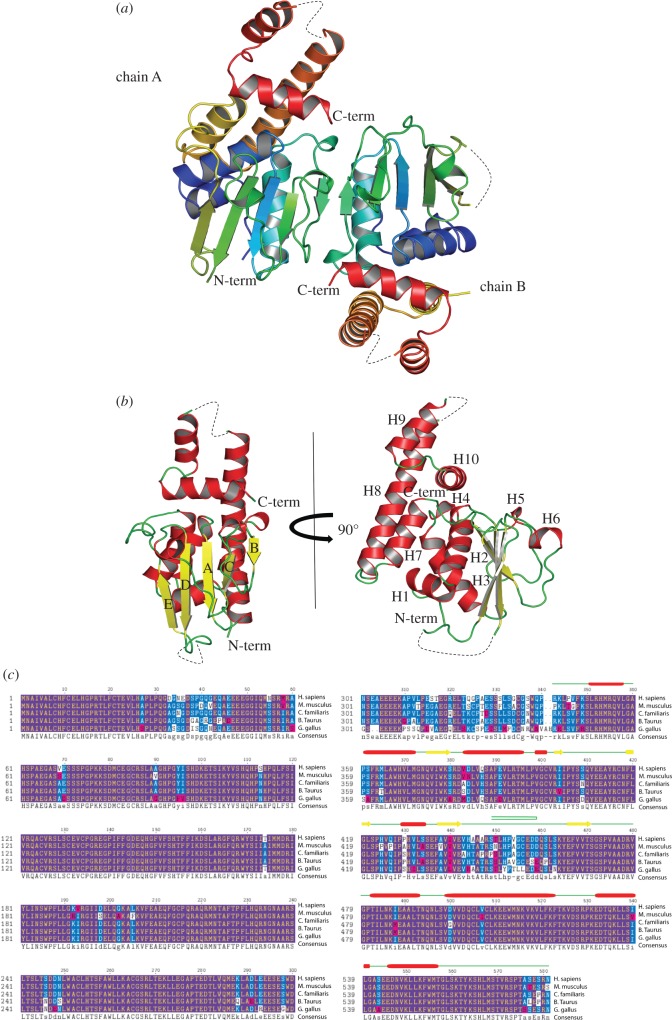
The crystal structure of folliculin-CT. (*a*) Crystal structure of the folliculin-CT presented at 2 Å resolution. The two molecules in the asymmetric unit are represented as a cartoon; chains A and B are rainbow coloured from blue at the N-terminus to red at the C-terminus. (*b*) The front view of the protein shows the arrangement of the β strands (labelled A–E) with the strand order B-C-A-D-E. The side view of the structure shows the majority of the ten helices (labelled H1–10) stacked onto the side of the protein. Dashed lines represent the loops not present in the crystal structure. (*c*) A TexShade representation of the alignment of folliculin protein sequences from higher vertebrates with the secondary structure of C-terminal domain overlayed. Highly conserved residues are shown as yellow letters in purple blocks. Conserved residues are shown as white letters in blue blocks. Semi-conserved residues are shown as white letters in pink boxes. The green lines represent the loops in the crystal structure, the red cylinders represent the helices and the yellow block arrows represent the β strands. The green U represents the hairpin in the structure.

In order to understand the essentiality of the C-terminal region (amino acids 341–566) and reveal the function of full-length folliculin, we have determined at 2 Å resolution the three-dimensional structure of the C-terminal domain of folliculin, henceforth called folliculin-CT. We have calculated phases by the multi-wavelength anomalous dispersion (MAD) method using anomalous scatterers from selenomethionine (see §4). The folliculin-CT crystallized in C222_1_ space group with two molecules in the asymmetric unit related by twofold non-crystallographic symmetry. The fold of folliculin-CT is dominated by an αβ architecture with a core β-sheet and helices packed on the one side, followed by an all-helical region (figure 1 and table 1). The NTPase αβ-domain comes high in Dali [18] structural searches of the Protein Data Bank, but this approach does not consider the connectivity between secondary structures. Indeed, the strand topology differs, and the signature Walker A and B motifs [19] conserved for function across NTPase families are absent in the folliculin-CT domain.

**Table 1.** X-ray data collection and refinement statistics for folliculin-CT.

	native	peak	inflection	remote
*data collection*				
X-ray source	Diamond, I03	Diamond, I02		
wavelength (Å)	0.98	0.9796	0.9797	0.9763
space group	C222_1_	C222_1_	C222_1_	C222_1_
cell dimensions				
a, b, c (Å)	86.05, 99.95, 107.58	88.94, 98.31, 109.40	88.65, 98.23, 109.31	88.37, 98.10, 109.28
α, β, γ (°)	90, 90, 90	90, 90, 90	90, 90, 90	90, 90, 90
resolution (Å)	29.85–1.92 (1.97–1.92)	65.95–2.7 (2.85–2.7)	65.81–2.7 (2.85–2.7)	65.66–2.7 (2.85–2.7)
*R*_sym_, %^a^	7.3 (64.8)	4.1 (78.2)	4.2 (56.5)	3.9 (45.3)
*I*/σ*I*	14.3 (2.1)	30.7 (3.6)	32.2 (4.7)	35.1 (5.8)
completeness (%)	97.6 (61.2)	100 (100)	99.7 (100)	100 (100)
redundancy	6.1 (4.5)	7.5 (7.2)	7.4 (7.1)	7.5 (7.2)
*refinement*				
resolution (Å)	29.14–2.0 (2.05–2.00)			
no. of unique reflections:				
total	29 744			
*R*_free_ set	1570			
*R*_cryst_/*R*_free,_ %^b,c^	20.4/26.8			
no. of atoms:				
protein	3078			
water	244			
average B-factor (Å^2^)	28.7			
r.m.s deviations:				
bond length (Å)	0.024			
bond angle (°)	1.925			

Values in parentheses show the corresponding statistics in the highest resolution shell.

^a^*R*_sym_ = *Σ*_h_|*I*_h_–<I>­ |/*Σ*_h_*I*_h_, where *I*_h_ is the intensity of reflection h, and <I> is the mean intensity of all symmetry-related reflections.

^b^*R*_cryst_ = *Σ*||*F*_obs_| − |*F*_calc_||/*Σ*|*F*_obs_|, where *F*_obs_ and *F*_calc_ are observed and calculated structure factor amplitudes.

^c^*R*_free_ as for *R*_cryst_ using a random subset of the data (about 5%) excluded from the refinement.

**Figure 2.**
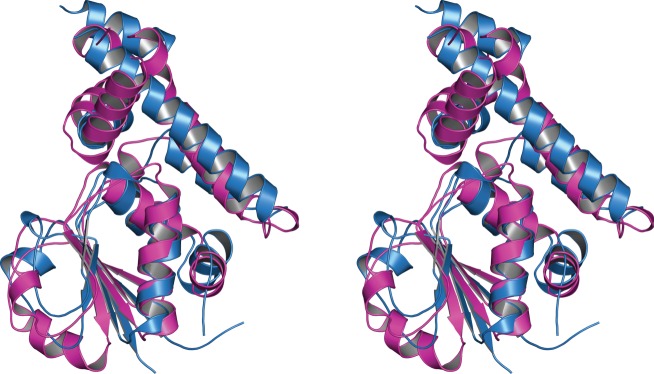
Folliculin-CT is structurally similar to the DENN domain of DENN1B. Walleye stereo image of the structural superposition showing the similarities of folliculin-CT and the DENN domain of DENN1B; the two protein chains are represented as a cartoon with folliculin-CT in blue and the DENN domain of DENN1B in magenta.

Recently, Wu *et al.* [20] have reported the crystal structure of a DENN domain containing protein with its cognate Rab GTPase, Rab35. The DENN domain family consists of a group of ancient but poorly understood proteins that share common structural features and have been shown to be GEFs for Rab GTPases [21]; they facilitate GDP–GTP exchange, thereby activating the Rab GTPase in vesicular transport [22,23]. Rab GTPases form the essential network of vesicle membrane transport both in exo- and endocytic pathways [22]. Interestingly, folliculin-CT shares remarkable structural similarity with the DENN domain of the DENN1B-S protein; both proteins have the same order and orientation of strands (figure 2). Although the sequence identity is only 11 per cent, a structural alignment using the program Baton (based on Comparer [24]) shows a r.m.s.d. of 2.8 Å over its 170 core-aligned residues, corroborating the strong similarity and probable homology. The amino acid conservation is evident in the Joy [25] alignment of 10 diverse homologues of both DENN1B and folliculin-CT (see the electronic supplementary material, figure S2).

**Figure 3.**
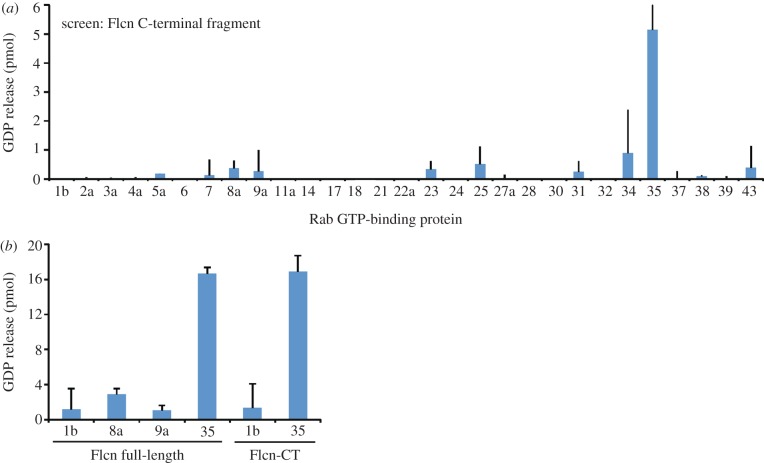
Folliculin possesses *in vitro* GEF activity towards Rab35. (*a*) The GEF assay screen performed with folliculin-CT on the Rab GTPase library. The GDP release is represented as a blue bar graph with error bars. (*b*) The same assay performed with full-length folliculin and folliculin-CT on the subset of Rabs that belong to the Rab 35 subfamily. Flcn, folliculin.

The structural similarity to the DENN domain protein DENND1B suggests that folliculin might possess GEF activity towards a member of the Ras-superfamily of small GTPases, most likely a Rab GTPase regulator of vesicular trafficking. Indeed, biochemical analysis of a representative set of Rabs reveals that folliculin-CT, *in vitro*, has GEF activity towards Rab35, and this is confirmed using a subset of Rabs with the full-length folliculin, which shows similar activity to the folliculin-CT (figure 3). These findings suggest that folliculin may act as a Rab GEF *in vivo*. Rab35 has been implicated in early endocytic trafficking, recycling events and cytokinesis [26–28]. Perturbed Rab35-dependent transport may lead to aberrant regulation of specific signalling pathways, separate from those regulated by Rab5 (canonical growth factor pathways, etc.; [29]). However, although the structural similarity to the DENND1B Rab GEF implicates a Rab and the *in vitro* data presented here suggest that Rab35 might be involved, the divergence of folliculins from the DENN family does not exclude involvement of other members of the 44 member human Rab family [21] or other Ras-like small GTPases.

Wu *et al.* [20] identified several conserved clusters of residues (within 5 Å radius of Rab35) within the DENN1 subfamily as being essential for Rab binding/GEF activity. However, there is little conservation of the sequences in these regions among the most diverged DENN proteins. In a similar way, the equivalent regions in folliculin are highly conserved within the close orthologues, but are not conserved in the most divergent folliculins. We conclude that, if functions are conserved, there must be complementary changes in the interacting DENNs and their partner GTPases. Therefore, until the physiologically relevant partner of folliculin is identified and a model of the interaction developed, it is not possible to test this hypothesis by mutagenesis of specific interacting residues.

An initial sequence-based classification of the DENN superfamily suggested that the DENN homology region is composed of three distinct modules: upstream or u-DENN, the better-conserved central or core or c-DENN, and downstream or d-DENN regions [21,24]. These modules have been lent a structural form by the DENN1B fold [30], with u-DENN mapping to an N-terminal longin domain, whereas the core DENN and d-DENN modules respectively match the α/β fold and helical tail extension. In order to gain further insights into the overall architecture of the full-length folliculin (and thereby understand its function), we used sensitive methods developed for structure-guided alignment of distant sequences [31], fold recognition and structure prediction [32,33], to build domain-level alignments of folliculin-like sequences, and to investigate the possibility of structural and functional similarities in the N-terminal and central regions of folliculin and the DENN superfamily. Indeed, folliculin is transitively linked to the DENN superfamily by HMM–HMM alignment [32] to both the core set of DENN sequences [34] as well as a more recently described, outlier branch of DENN proteins related to yeast AVL9, which functions in exocytic transport [35].

The truncated folliculin polypeptide chain found in BHD syndrome is missing the c-DENN and d-DENN modules, and retains only the u-DENN region—which is the only part of the DENN homology region that is kept in the more compact yeast folliculin orthologue, LST7. The predicted u-DENN region in folliculin is linked by an approximately 40+ amino acid disordered segment to the Rab-interacting c-DENN:d-DENN modules; the equivalent linker was removed (for crystallographic purposes) in the structure described for DENN1B [20]. In folliculin, this connector region has a stretch of acidic residues. It also harbours a bipartite tryptophan (WD–WQ) motif, which has been shown to be a kinesin light chain 1 interacting motif [36]; interestingly, the DENN1B protein (isoform 5) has a similar motif in the region spanning residues 629–729 [36].

**Figure 4.**
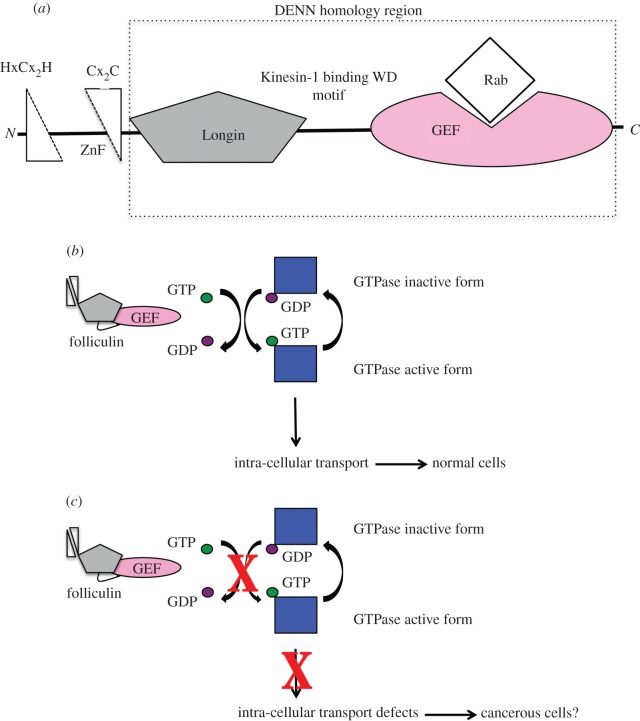
Putative domain architecture of folliculin and a possible mechanism for folliculin GEF activation of its Rab. (*a*) Schematic of the putative domain organization of folliculin. The N-terminal zinc-binding domain is represented as right-angled triangles. The grey pentagon represents the longin domain. The kinesin light chain 1 binding bipartite tryptophan motif is represented as a line. The GEF domain is represented as a pink oval. (*b* and *c*) Schematic of the possible mechanism of interaction between folliculin and its cognate GTPase.

Given the probable distant homology between folliculin and the larger DENN superfamily, and the close structural resemblance between folliculin and DENN1B protein (the Rab binding c-DENN:d-DENN modules and linker bipartite tryptophan motif), the N-terminal regions of both proteins were scrutinized for closer architectural similarities. The N-terminal 85 amino acids of folliculin comprise a conserved HxCx_2_H-{28–56 residues}-Cx_2_C putative zinc-ion-binding module (figure 4*a*), which is absent in the DENN superfamily. However, the following 160 amino acids have a predicted secondary structure pattern [32,37] that suggestively matches that of the prototypic longin domain fold (with a ββαβββαα topology [34]) found in the DENN1B N-terminal module. Although in folliculin there is a long loop predicted between the two C-terminal helices, HHpred [32] significantly aligns this folliculin segment with the DENN1B longin domain, suggesting that the N-terminus of folliculin might encompass a divergent longin-like fold, preceded by a split zinc-binding domain (figure 4*a*). Currently, efforts are underway to determine the crystal structure of this region. The presence of a longin domain would settle folliculin's kinship with the DENN superfamily, and would have implications for its function in regulation of membrane trafficking [38], as well as in structural support—*per* DENN1B—of Rab GEF function.

Genetically inherited syndromes offer an important insight into molecular mechanisms underlying biological processes of medical importance, such as carcinogenesis. Thirty-five years after the initial description of the inherited skin lesions and the subsequent association with the folliculin gene, we present data that address the molecular mechanism of BHD syndrome. Through structure–function analyses, we report that folliculin, which is commonly mutated in patients suffering from these malignancies, has an unusual architecture that classes it into an ancient family of proteins called the DENNs. Because most BHD symptoms are attributed to a truncated folliculin protein or its complete absence, we infer that the loss of folliculin and therefore lack of its cognate GTPase activation might be compromising the latter's regulatory function in membrane trafficking (figure 4*b*,*c*). Given the severity of the phenotype in BHD syndrome, we suggest that folliculin's GEF activity towards its GTPase might be essential for important cellular processes. In this context, it would be interesting to investigate defective endocytic transport in BHD patients.

## 4. Methods

### 4.1. Folliculin protein purification

The folliculin-CT was cloned into the Gateway system (Invitrogen, UK), and the recombinant protein was expressed as a thioredoxin fusion protein in BL21 (DE3) Star (Invitrogen, UK) *Escherichia coli* competent cells. The recombinant protein was initially purified over a 1 ml nickel-immobilized metal affinity chromatography column (Ni-IMAC). A proteolytic cleavage using tobacco etch virus protease was carried out for 1 h at room temperature to separate the affinity tag from folliculin-CT. The reaction was subsequently passed over the Ni-IMAC column for removal of the tag and the remaining uncleaved fusion protein. The target protein was further purified using a Superdex 75 (GE Healthcare) size exclusion chromatography column to obtain homogeneous recombinant protein for crystallization trials. The protein was concentrated to 5 mg ml^−1^ using Amicon ultra filter concentrators (Millipore, UK) with a 10 000 daltons molecular weight cut-off membrane. The concentrated protein was frozen in liquid nitrogen and stored at −80°C until further use. Full-length folliculin was cloned into pOPINS (a generous gift from Dr Roger Dodd) and the recombinant protein was expressed as a SUMO fusion protein in BL21 (DE3) Star (Invitrogen, UK) *E. coli* competent cells. The recombinant protein was initially purified over a 6 ml Ni-IMAC. The resulting elution fractions were treated with 5 mM MgCl_2_ + 5 mM ATP, and the target protein was further purified using a Resource Q (GE Healthcare) ion exchange column. Proteolytic cleavage of the recombinant protein was performed using SUMO protease. The protein was then passed again through the Ni-IMAC, to remove the uncleaved protein and the fusion tag. The protein was concentrated to 60 μM using Amicon ultra filter concentrators (Millipore, UK) with 30 000 daltons molecular weight cut-off membrane. The concentrated protein was snap frozen in liquid nitrogen and stored at −80°C until further use.

### 4.2. Mutagenesis

Initial crystallization attempts with folliculin-CT wild type (corresponding to the region 341–579 aa) resulted in no significant crystals. Crystallization of the folliculin-CT required mutation of three cysteine residues, Cys 454, Cys 503 and Cys 506, to alanines. These cysteine residues could have been forming covalent intermolecular disulphide-mediated cross-links that were causing folliculin-CT to form multimers thereby inhibiting crystallization. Furthermore, the terminal 13 amino acids (predicted to be disordered) were removed by introducing a stop codon after the residue 566 to prevent the protein from degradation. Mutagenesis of the cysteine residues to alanines in folliculin-CT was carried out using the Quick Change mutagenesis kit (Stratagene), according to the manufacturer's protocol. For mutagenesis of folliculin-CT, the PCR mixture contained 1 μl of template DNA (5 ng and 25 ng), 1 μl of forward primer (10 μM), 1 μl of reverse primer (10 μM), 1 μl of dNTPs (100 mM), 1 μl of P*fu* turbo polymerase (2 U), 5 μl of 10× reaction buffer and 40 μl of Milli Q water. The PCR cycles were composed of one cycle of 95°C for 30 s and 16 cycles of 95°C for 30 s, 55°C for 1 min and 68°C for 8 min 20 s. Following PCR, the reactions were treated for 1 h with 1 μl *Dpn*I restriction enzyme at 37°C to remove methylated DNA. Subsequently, 1 μl of the treated reaction was transformed into XL1-Blue Super-competent *E. coli* and plated onto LB agar plates with 100 μg ml^−1^ of ampicillin. The plates were incubated overnight at 37°C to facilitate the growth of bacterial colonies. Several colonies from each plate were sub-cultured into 5 ml LB broth medium with ampicillin. The DNA from the cultures was extracted using Qiagen Miniprep kit and the DNA sequence was verified for the desired mutations. The following primers were used for mutagenesis: Cys 454 Ala forward primer CTC CAC CCT GTG GGG GCT GAG GAT GAC CAG TCT and Cys 454 Ala reverse primer AGA CTG GTC ATC CTC AGC CCC CAC AGG GTG GAG; Cys 503, 506 Ala forward primer GAT GTG GTG GAC CAG GCC CTC GTC GCC CTC AAG GAG GAG TGG and Cys 503, 506 Ala reverse primer CCA CTC CTC CTT GAG GGC GAC GAG GGC CTG GTC CAC CAC ATC.

### 4.3. Crystallization

Initially, the crystallization trials were set up with 5 mg ml^−1^ folliculin-CT using 96 well crystallization plates (Griener, UK) and JCSG Crystal Screen (Molecular Dimensions, UK) and incubated at 12°C. Initial crystals appeared overnight in condition H9, which corresponds to 0.2 M LiSO_4_, 0.1 M Bis–Tris pH 5.5 and 25 per cent PEG 3350 (henceforth referred to as the ‘mother liquor’). The crystals were reproduced by optimizing the mother liquor using the vapour diffusion method in 24-well Linbro plates. Crystals were harvested in cryo-protectant solution containing 30 per cent polypropylene glycol (PPG; Sigma, UK) along with the original mother liquor using crystallization loops (Hampton Research Inc, USA), and stored in liquid nitrogen.

### 4.4. Data collection, phasing and structure refinement

Initial structure of folliculin-CT was determined by collecting MAD from selenomethionine crystals at Diamond Light source beam line I02, exploiting the anomalous signal of the incorporated selenium. Three data sets corresponding to the peak, inflection and remote energies for selenium atoms were acquired on a single folliculin-CT crystal. The data for selenium peak were collected at 12 657.42 eV (**λ** = 0.9796 Å), the inflection data were collected at 12 656.42 eV (**λ** = 0.9797 Å) and finally the remote data were collected at 12700.42 eV (**λ** = 0.9763 Å). The diffraction data were processed in iMosflm (S1) and merged and scaled in Scala (S2) of CCP4i (S3). The positions of the selenium atoms in the asymmetric unit were determined using Autosol wizard of Phenix (S4) software package. Fourteen selenium atom sites (seven from each molecule in the asymmetric unit) were identified and the resulting figure of merit after density modification was 0.64. The solvent-modified map calculated by Autosol was interpreted by Autobuild wizard, which produced an almost complete model of the structure. Refinement of the structure was carried out in Refmac5 (S5), together with manual protein structure rebuilding in COOT (S6). It was observed that the electron density was not very clear or totally absent in the following regions: residues 341–343 in both chain A and B; residues 443–458 (in chain A) and 447–459 (in chain B); residues 469–477 in chain B only; residues 523–528 (chain A) and 523–527 (chain B); residues 557–566 (chain A and chain B). Hence, it was not possible to build these regions. Subsequently, higher resolution data to 1.92 Å resolution using native protein crystals were collected at Diamond Light Source beam line I03. The final structure of folliculin-CT was obtained by the molecular replacement method using Phaser_MR (S7) module of CCP4i where the original model built at 2.7 Å was used as the molecular replacement search probe. The model was then refined and manually adjusted using the programs mentioned earlier. For refinement calculations, the resolution of the native dataset was truncated to 2.0 Å as the higher resolution shell had completeness of less than 70 per cent. To improve the electron density maps the refined structure was used to generate a four-segment TLS (translation/libration/screw) model using the TLSMD server (TLS motion determination; (S8,S9)). The resultant TLS parameters were used in further refinement to obtain the final *R*/*R*_free_ values of 20.4/26.8 per cent, respectively. The final model has Ramachandran statistics showing that 375 residues are present in the preferred regions (98.68%), five residues in allowed regions (1.32%) and no residues in disallowed regions. All structure figures were generated using PyMOL (S10).

### 4.5. Guanine nucleotide exchange assay

Nucleotide loading was carried out as follows: 10 µg GST-tagged Rab was incubated in 50 mM HEPES–NaOH pH 6.8, 0.1 mg ml^−1^ BSA, 125 µM EDTA, 10 µM Mg-GDP and 5 µCi [^3^H]-GDP (10 mCi ml^−1^; 5000 Ci mmol^−1^) in a total volume of 200 µl for 15 min at 30°C. For standard GDP-releasing GEF assays, 100 µl of the loading reaction was mixed with 10 µl 10 mM Mg-GTP, 10–100 nM GEF protein to be tested or a buffer control, and adjusted to 120 µl final volume with assay buffer. The GEF reaction occurred for 20 min at 30°C. The reaction was split into two tubes, then incubated with 500 µl ice-cold assay buffer containing 1 mM MgCl_2_ and 20 µl packed glutathione–sepharose for 60 min at 4°C. After washing three times with 500 µl ice-cold assay buffer the sepharose was transferred to a vial containing 4 ml scintillation fluid and counted. The amount of nucleotide exchange was calculated in pmoles of GDP released.

## 5. Acknowledgements

The authors thank Dr Julia Forman for carrying out initial bioinformatic analyses. We are grateful to Dr Mark Dodding, King's College London for discussion of WD motifs. We thank Dr Len Packman and Mr Michael Weldon in the PNAC facility at the Department of Biochemistry, University of Cambridge for help with mass spectrometry analysis and protein N-terminal sequencing of folliculin samples, respectively. The crystallographic experiments were performed in the X-ray crystallographic facility at the Department of Biochemistry, University of Cambridge. We are grateful to the facility manager, Dr Dimitri Chirgadze, for his assistance in using these facilities. We thank Mr John Lester for help with DNA sequencing of folliculin clones. Folliculin cDNA was a generous gift from Prof. Eamonn Maher at University of Birmingham. The authors thank the Myrovlytis Trust for funding to R.K.N, A.P. and B.K.B. B.O-M is supported by a grant from the Bill and Melinda Gates Foundation. J.C.D is funded by a BBSRC studentship. L.L, F.A.B., D.Y.C and T.L.B. are funded by the Wellcome Trust. R.K.N and T.L.B conceived and designed the experiments. R.K.N expressed, purified, crystallized and determined the three-dimensional structure of the recombinant folliculin-CT. J.C.D. cloned and expressed the initial constructs of folliculin domains. D.Y.C. helped in structure building, refinement and analysis. J.F.B. performed the bioinformatics analyses on DENN domains along with A.P. and B.O-M. A.P. purified the full-length folliculin for GEF assays. B.K.B. performed mutagenesis on folliculin-CT constructs and purified folliculin-CT mutant proteins. L.L. and F.A.B. carried out the GEF assays to identify folliculin's Rab. All authors contributed to the writing of the manuscript. The atomic coordinates and structure factors for folliculin-CT crystal structure have been deposited with the Protein Data Bank under accession code 3V42. The authors declare no competing financial interests.

## Supplementary Material

Supplementary Figures
