# Supplementary material for: Crystal structure of folliculin reveals a hidDENN function in genetically inherited renal cancer
Source: Open Biol. 2012 Aug;2(8):120071. doi: 10.1098/rsob.120071 (PMC3438538; doi:10.1098/rsob.120071)
Supplement: Supplementary Figures [file rsob120071-s1.doc]

**Supplementary Figures**

**
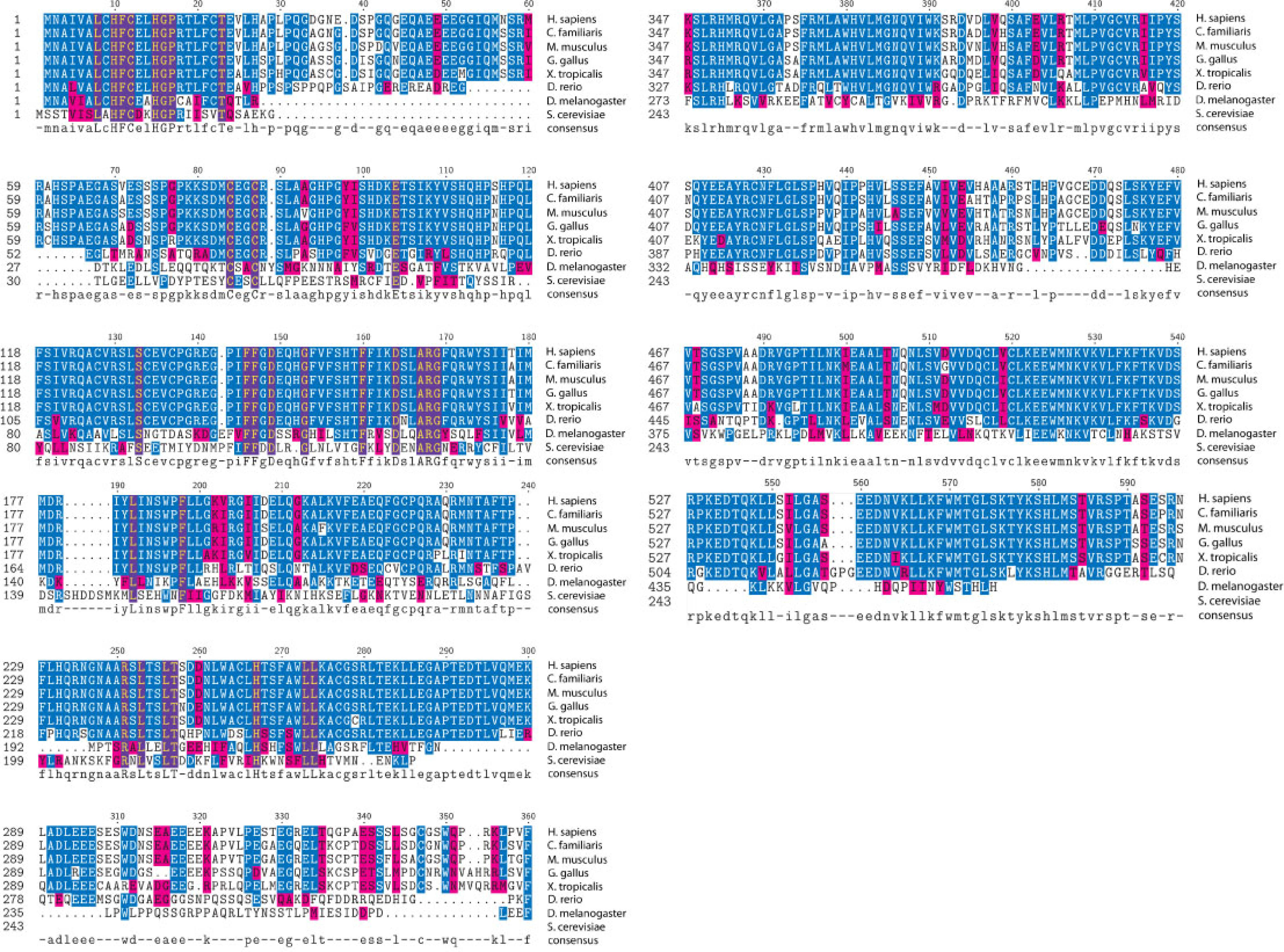
**

**Supplementary Figure 1. Sequence alignments of folliculin from various genera.** A TexShade representation of the alignment of various folliculin protein sequences showing the evolutionary conservation.Highly conserved residues are shown as yellow letters in purple blocks.Conserved residues are shown as white letters in blue blocks. Semi-conserved residues are shown as white letters in pink boxes.

**
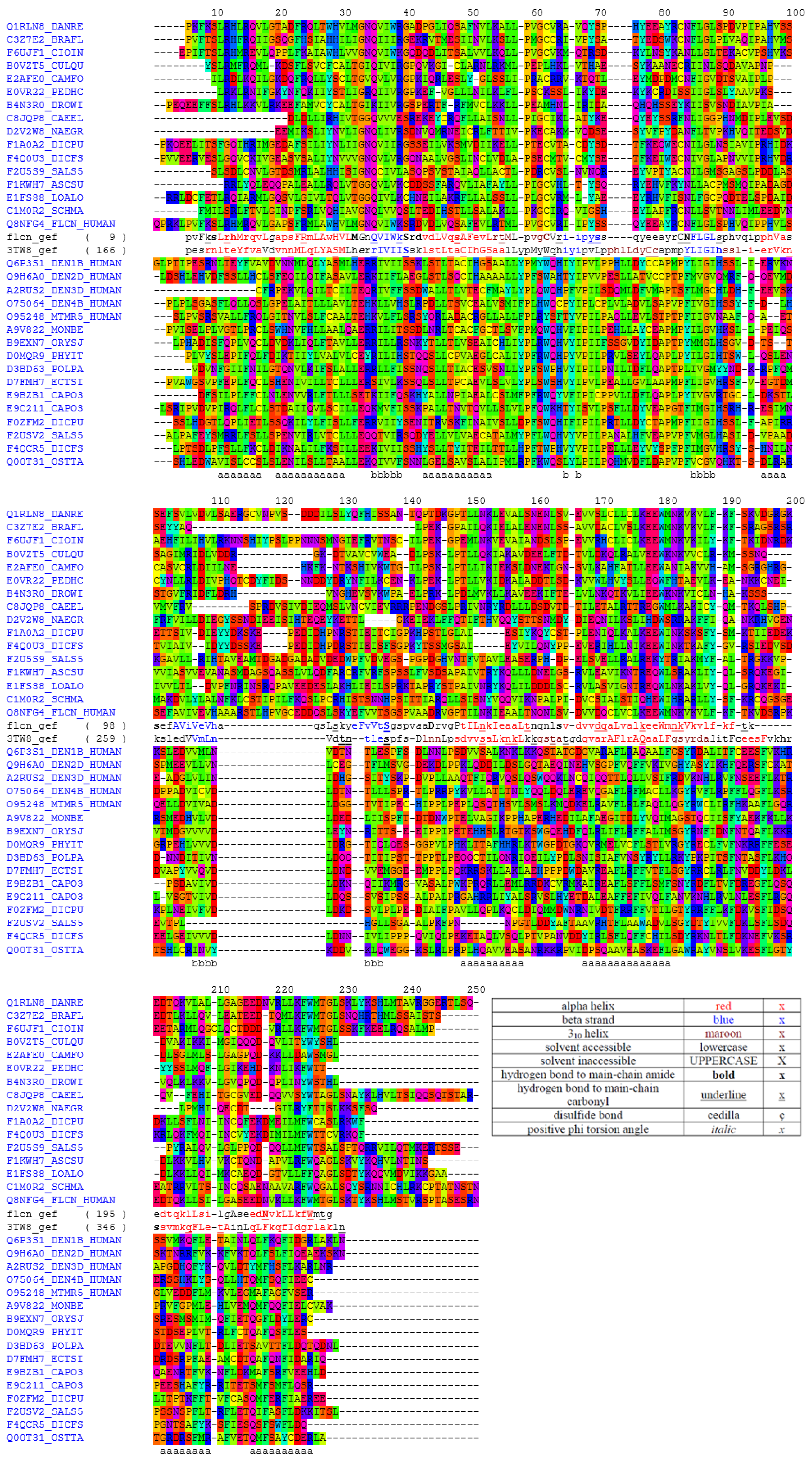
Supplementary Figure 2. Structural alignment of folliculin-CT with the GEF domain of DENN1B**. Fifteen diverse homologs each of folliculin and DENN domain proteins aligned to their sequence, without inserts. The structures were aligned using BATON^1^ with manual refinements and annotated using JOY (key in box; [16]). The homologue sequences, labelled with their UniProt identifier and species code, were obtained using PSI-BLAST [S11] on UniRef90 [S12] database and realigned using T-COFFEE [S13] consensus method with MAFFT [S14], Dialign-TX [S15], Muscle [S16] and ProbCons [S17] algorithms. Residues are coloured according to Taylor’s scheme.

**
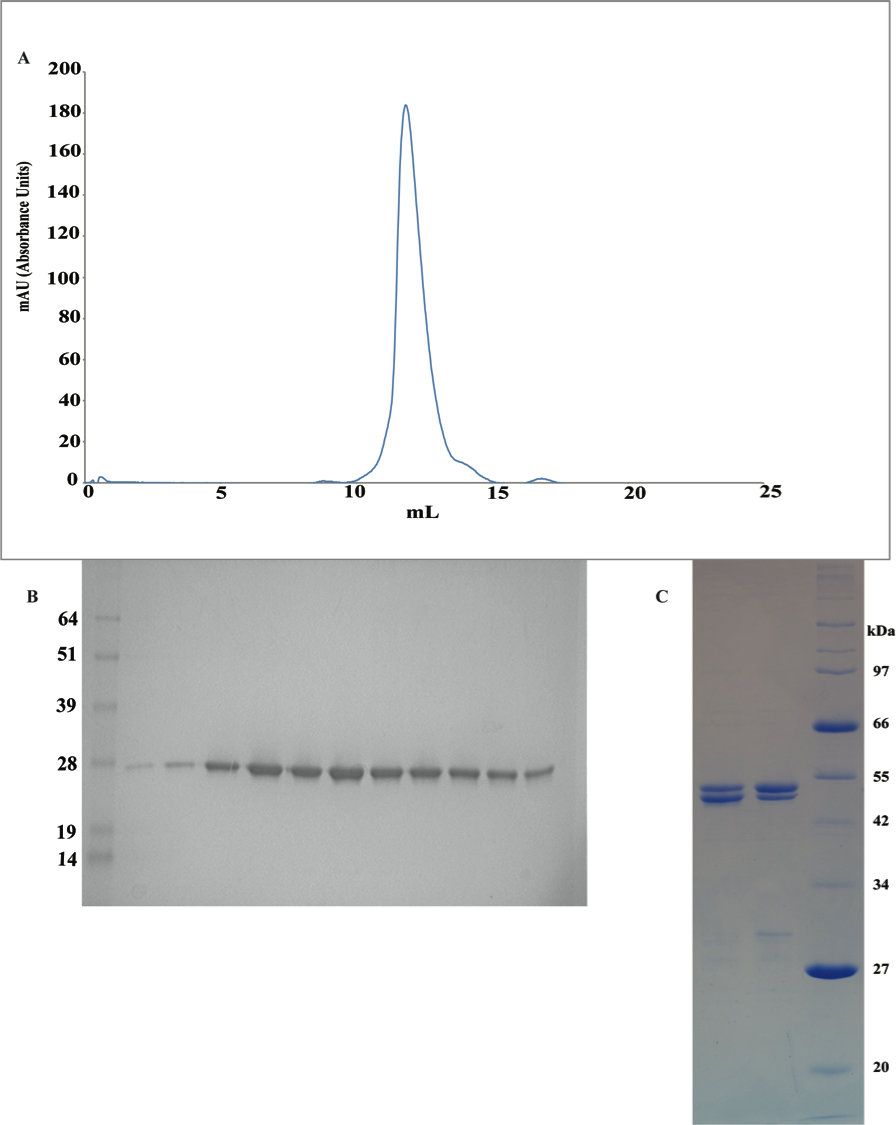
**

**Supplementary Figure 3. SDS-PAGE and Size exclusion chromatography analyses of folliculin-CT. A)** A profile of size exclusion chromatorgraphy using a Superdex 75 resin based column of folliculin-CT showing that the protein migrates as a monomer. **B)**  SDS-PAGE anaylses showing the purity of the folliculin-CT protein. **C)** An represenatative example of the SDS_PAGE analyses of purified Rab proteins used in GEF assays.
